# Correlation between severity of ultrasonographic nonalcoholic fatty liver disease and cardiometabolic risk among Filipino wellness patients

**DOI:** 10.15171/jcvtr.2017.14

**Published:** 2017-06-08

**Authors:** Lucky R. Cuenza, Tamara Louise J. Razon, Juan Carlo Dayrit

**Affiliations:** ^1^Section of Preventive Cardiology and Cardiac Rehabilitation, Philippine Heart Center, Manila, Philippine; ^2^Department of Radiology, The Medical City Clark, Pampanga, Philippine; ^3^Medical Center Manila, Manila, Philippine

**Keywords:** Cardiometabolic, Nonalcoholic Fatty Liver Disease, Framingham Risk Score

## Abstract

***Introduction:*** Nonalcoholic fatty liver disease (NAFLD) is a prevalent condition which is known to be related to factors that predispose to the development of coronary artery disease as well as development of metabolic syndrome. The study aimed to determine the association between ultrasound-based grading of hepatic steatosis with metabolic profile and estimated cardiovascular risk using the Framingham Risk Score (FRS).

***Methods:*** This was a cross-sectional study on 100 Filipino patients without established cardiovascular disease who underwent a general wellness health evaluation. Cases with NAFLD diagnosed on the basis of ultrasound grading were analyzed. Comparison of demographics and metabolic parameters between grades of hepatic steatosis was performed using Kruskal Wallis test. FRS was used to assess cardiovascular risk with Spearman rank test for correlation with the degree of NAFLD.

***Results:*** Mean age was 47 ± 9.6 years, with 70% males. Mean body mass index (BMI) was 28.7 ± 5.1. Most patients had grade I NAFLD (53%), 34% were grade II, and 13% were grade III. BMI (*P *=0.034), liver enzymes (alanine aminotransferase [ALT], *P *= 0.001; aspartate aminotransferase [AST], *P *= 0.00), triglycerides (*P *= 0.047), and fasting blood sugar [FBS] (*P *= 0.049) were associated with fatty liver grade. No association was noted with total cholesterol (*P *= 0.569), high density lipoprotein (HDL) (*P *= 0.220), and low density lipoprotein (LDL) (*P *= 0.792). Using the FRS 43% were stratified as low (<10% risk), 45% as intermediate (10%-20% risk) and 12% as high risk (>20% risk). Severity of fatty liver was directly correlated with the FRS (Spearman rank 0.741, *P *= 0.009).

***Conclusion:*** Ultrasound-based grading of the severity of NAFLD is associated with abnormalities in the metabolic profile of patients. The FRS is correlated with increasing severity of NAFLD based on ultrasound. These findings suggest that the presence of NAFLD may be a marker for the presence of increased cardiovascular risk and may help identify patients who may benefit from more aggressive therapies to prevent development of adverse cardiovascular events.

## Introduction

Nonalcoholic fatty liver disease (NAFLD) was previously defined by Ludwig in 1980 describing the biopsy findings in patients with steatohepatitis in the absence of significant alcohol consumption.^1^ NAFLD encompasses a spectrum of liver damage, ranging from simple or bland steatosis to nonalcoholic steatohepatitis (NASH) associated with progressive liver damage and complications ranging from fibrosis, cirrhosis, and liver cancer.^2^ NAFLD classification system may have correlation with certain histological features that are related to long term prognosis. Grade I pertains to simple steatosis; grade II is steatosis accompanied with lobular inflammation and ballooned hepatocytes; and, grade III combines all these features with fibrosis.^3^

The reported prevalence of NAFLD in the United States is about 5% of the general population, reaching 25% to 75% in patients with obesity and type 2 diabetes mellitus. A local study in a tertiary center noted a prevalence of 12.2% in the Philippines.^4^ NAFLD has been associated with metabolic syndrome and its components of obesity, hypertension, dyslipidemia, and insulin resistance, and an increased risk of atherosclerotic coronary heart disease (CHD) and diabetes.^5^

Recognition of the importance of NAFLD and its association with the metabolic syndrome and other cardiovascular risk factors made us to identify this correlation in our patient population. Accumulating evidence suggests that the link between these two disorders involves components of atherogenesis and inflammation and may play a role in patient outcomes and treatment.^6^

The reported sensitivity of ultrasonography in detecting hepatic steatosis ranges from 60% to 94% and the specificity from 84% to 95%.^7^ Compared to liver biopsy ultrasound provides a non invasive way of determination of the degree of severity of NAFLD. The diagnosis and determination of the degree of steatosis using this modality may be correlated with the metabolic profile^8^ of patients as well as the degree of cardiovascular risk. No study in our local setting has yet been performed examining this correlation. We aimed to determine the correlation of the degree of ultrasonographic fatty liver severity with the cardiometabolic profile of patients.

## Materials and Methods

This was a cross sectional study of 100 patients undergoing an executive check-up package at the Corporate Health and Wellness Clinic and the Radiology Department Ultrasound Section of the Makati Medical Center. The wellness package consists of a general cardiometabolic screening with inclusion of liver ultrasound. Patients diagnosed with NAFLD based on history and ultrasound imaging were included. Patient records including clinical history, physical exam, and ancillary and laboratory test results were reviewed. Male and female patients 18 years of age and above were included in the study only after taking informed consent from the patient.

Exclusion criteria were as follows: patients with established liver disease or focal hepatic lesions such as hepatitis, cirrhosis, or nodules/masses, a history of significant alcohol consumption, defined by more than 30 g/d in females and 20 g/d in males, and pregnancy. Patients with any history of taking drugs or supplement that may influence the lipid profile and liver enzymes or may induce or reduce fatty liver changes were also excluded.

High-resolution gray scale B-mode transabdominal ultrasound imaging was utilized in the diagnosis of fatty liver disease. The ultrasound procedure was performed by the sonologist and the radiologist on deck, who was blinded to the patient characteristics, interpreted the images. The imaging-based grading of NAFLD was facilitated by a review of the official ultrasound reports as well as stored digital images in the picture archiving and communication system (PACS). All images and ultrasound reports were verified by one of the co-authors (TR). Criteria used for grading is classified as follows: Grade I (mild) - increased parenchymal echogenicity with visible periportal and diaphragmatic echogenicity; Grade II (moderate) - increased parenchymal echogenicity with obscuration of the echogenic walls of the portal vein branches, without obscuration of the diaphragm and Grade III (severe) - increased parenchymal echogenicity with imperceptible periportal echogenicity and obscuration of the diaphragmatic outline.

Patient records were reviewed for both demographic and metabolic profiles. Parameters that were assessed included: age; gender; body mass index (BMI); liver enzymes namely alanine aminotransferase (ALT) and aspartate aminotransferase (AST); lipid profile including triglycerides, total cholesterol, high density lipoprotein (HDL) and low density lipoprotein (LDL); and, fasting blood sugar (FBS).

Using patient variables (age, gender, total cholesterol, HDL, systolic blood pressure, on hypertension medications, presence of diabetes, smoking status) The Framingham Risk Score (FRS) was calculated to determine the degree of cardiovascular risk.9 Patients with an estimated 10 year risk of myocardial infarction or cardiovascular death of <10% were classified as low risk, 10%-20% as moderate or intermediate risk and >20% as high risk.

### Data analysis

Categorical variables were presented as frequency and percentage, and continuous variables were presented as mean and standard deviation (SD). The variables were compared by using a Kruskal-Wallis test with multiple comparison tests. The correlation of the FRS with the degree of hepatic steatosis was done using Spearman rank correlation test. A P value of <0.05 was considered significant. All statistical analyses were performed using SPSS 15 (SPSS Inc., Chicago, IL)

## Results

The baseline characteristics of the patients are listed (Table 1). The mean age of the study population is 47 ± 9.6 years, with 70% comprised of the male gender. The mean BMI is 28.7 ± 5.1 kg/m2. Results of the lipid profile reveal that most of the subjects have an elevated total cholesterol level (207.3 ± 51.9 mg/dL) and LDL level (143.6 ± 47.9 mg/dL). The mean triglyceride level is 160.2 ± 97.7 mg/dL. The mean FBS level is 106 ± 33.2 mg/dL. The mean ALT and AST levels are 35.4 ± 18.9 U/L and 25 ± 7.2 U/L, respectively.

**Table 1:** Baseline Characteristics

**Baseline characteristics**	**Total** **No. (%) or ±SD**
Male gender	70 (70%)
Age (y)	47 ± 9.6
BMI (kg/m^2^)	28.7 ± 5.1
ALT (U/L)	35.4 ± 18.9
AST (U/L)	25.0 ± 7.2
Triglycerides (mg/dL)	160.2 ± 97.7
Total cholesterol (mg/dL)	207.3 ± 51.9
HDL (mg/dL)	52 ± 21.9
LDL (mg/dL)	143.6 ± 47.9
FBS (mg/dL)	106 ± 33.2
Smoking	24 (24%)

Abbreviations: BMI, body mass index; FBS, fasting blood sugar; HDL, high-density lipoprotein; LDL, low-density lipoprotein.

Majority of the patients (53%) were classified as grade I steatosis on ultrasound, 34% were grade II, and 13% are grade III (Table 2).

**Table 2:** Degree of hepatic steatosis

**Grade**	**No. (%)**
I - Mild	53 (53)
II - Moderate	34 (34)
III - Severe	13 (13)
Total	100

Table 3 shows the correlation of the severity of the ultrasound-based grade of hepatic steatosis with the different metabolic parameters. An increasing BMI is correlated with an increasing severity of steatosis (*P* = 0.034). Likewise, there is a significant correlation of the levels of liver enzymes with the grade of steatosis (ALT, *P* = 0.001; AST, *P* = 0.000). Similarly, triglyceride levels are highest among Grade III patients (187.7 ± 61.6 mg/dL) compared to Grade II (180 ± 38.7 mg/dL) and Grade I (140.74 ± 65.28) patients, and the differences are statistically significant (*P* < 0.047). FBS levels are positively correlated with increasing severity (*P* = 0.049). Majority of the patients have elevated total cholesterol (*P* = 0.569), HDL (*P* = 0.220), and LDL (*P* = 0.792) levels. However, no correlation was noted between these parameters and grade.

**Table 3:** Correlation of severity of ultrasound-based grading of hepatic steatosis with metabolic parameters

**Parameter**	**Grade I**		**Grade II**		**Grade III**		***P*** ** value**
	**Mean**	**SD**	**Mean**	**SD**	**Mean**	**SD**	
AGE	‏47	‏±‏ 10.9	‏48	‏±‏ 8.2	‏44	‏±‏ 7.0	‏0.353
BMI	‏27.4	‏±‏ 3.2	‏29.2	‏±‏ 4.7	‏32.5	‏±‏ 9.3	‏0.034*
ALT	‏31.03	‏±‏ 18.83	‏36.5	‏±‏ 14.9	‏50.3	‏±‏ 22.1	‏0.001*
AST	‏22.8	‏±‏ 6.0	‏25.4	‏±‏ 6.2	‏32.6	‏±‏ 9.3	‏0.000*
TRIG	‏140.7	‏±‏ 65.2	‏180	‏±‏ 38.7	‏187.7	‏±‏ 61.6	‏0.047*
TCHO	‏205.6	‏±‏ 53.1	‏214	‏±‏ 52.1	‏196.8	‏±‏ 48	‏0.569
HDL	‏55.4	‏±‏ 27.5	‏49.1	‏±‏ 13.1	‏45.5	‏±‏ 8.1	‏0.220
LDL	‏142.9	‏±‏ 51.1	‏147.3	‏±‏ 44.5	‏136.8	‏±‏ 46	‏0.792
FBS	‏106.5	‏±‏ 32.7	‏101.8	‏±‏ 23.1	‏114.6	‏±‏ 53.3	‏0.049*

Abbreviations: BMI, body mass index; TRIG, triglycerides; TCHO, total cholesterol; FBS, fasting blood sugar; HDL, high density lipoprotein; LDL, low density lipoprotein.

**Figure 1:**
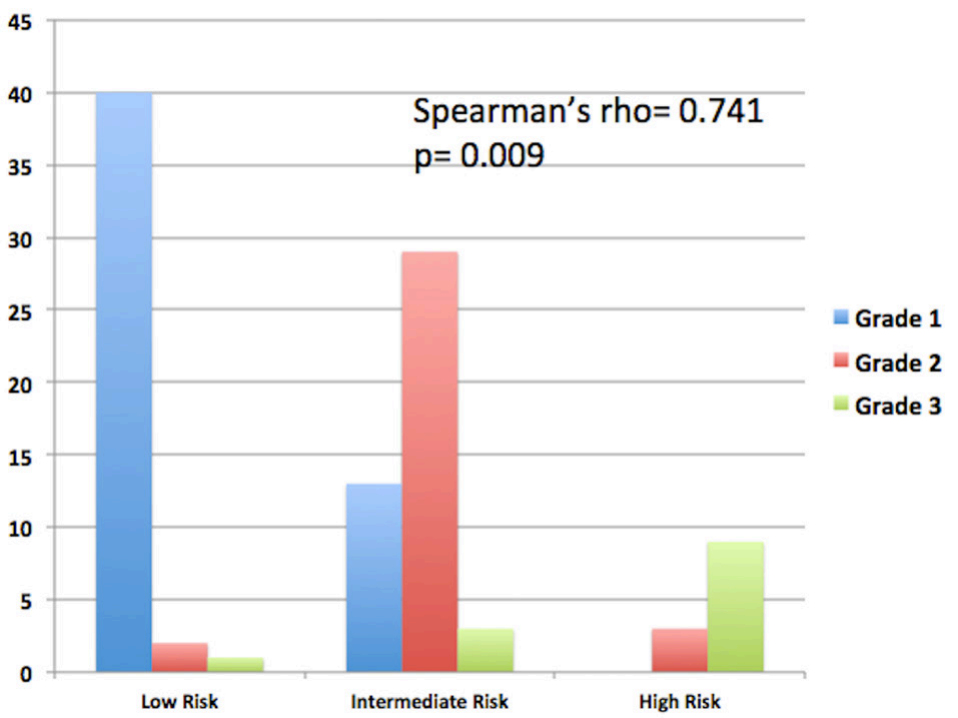
Estimated cardiovascular risk stratification categorized by severity of ultrasonographic grade of NAFLD

According to cardiovascular risk stratification using the FRS, 43 patients (43%) were in the low risk category, 45 (45%) in the moderate risk and 12 patients (12%) were classified as high risk, respectively (Table 4). Figure 1 compares the frequency of risk stratification with the degree of NAFLD. In the low risk group, there were 40 patients with Grade 1 NAFLD, 2 had grade 2 while only 1 patient had grade 3. In the intermediate risk group, 13 were classified with grade 1 steatosis, 29 had grade 2 and 3 patients had grade 3 NAFLD. For patients in the high risk category, none had grade 1 NAFLD, 3 patients had grade 2 and 9 patients had grade 3 NAFLD. Spearman rank analysis shows a significant correlation between the Framingham risk category and the degree of NAFLD. (Spearman rho=0.741, *P* = 0.009).

**Table 4:** Distribution of patients based on Framingham risk category

**Framingham Risk Score**	**Total** **No. (%) or ±SD**
Low (<10%)	43 (43)
Intermediate (10-20%)	45 (45)
Severe (>20%)	12 (12)
Mean FRS	11.1 ± 6.1

## Discussion

NAFLD is increasingly being recognized as a major cause of liver related morbidity and mortality. NAFLD can result in end stage liver disease, is implicated in some cases of cryptogenic cirrhosis, and has been proposed to lead to hepatocellular carcinoma.^10^ There is a well established association between NAFLD and metabolic syndrome. There is an increasing prevalence of metabolic syndrome, which is associated with obesity, dyslipidemia, and insulin resistance, among other cardiovascular risk factors.^11^

The positive correlations of higher range of BMI, elevated serum triglycerides, and evidence of impaired fasting glucose in patients diagnosed with NAFLD reflect a trend towards a predisposition to metabolic syndrome. It has been demonstrated that NAFLD occurs in approximately 20% of obese patients and 5% of overweight patients, and has a 2.6 fold association with diabetes.^12^ Obesity is present in the majority of individuals with NAFLD and was noted to be an independent risk factor strongly associated with the progression of the disease.^13^ Another study likewise showed that subjects with NAFLD exhibit higher triglyceride levels the accumulation of which is a consequence of saturation of fatty acid oxidation and increased secretion of VLDL.^14^

In the study, the mean HDL level is within the normal range at 52 ± 21.9 mg/dL. The mean total cholesterol level (207.3 ± 51.9 mg/dL) and mean LDL level (143.6 ± 47.9 mg/dL) are both above the normal range. However, these three parameters did not correlate significantly with the severity of fatty liver disease on ultrasound-based grading. Similarly, another study noted that biopsy proven NAFLD correlated with hepatic inflammation but did not correlate with lipid parameters.^15^ This lack of a positive correlation with cholesterol levels may be attributed to the complex nature of lipid metabolism. The primary metabolic abnormalities leading to lipid accumulation are not well understood, but may consist of alterations in the pathways of uptake, synthesis, degradation, or secretion in hepatic lipid metabolism resulting from insulin resistance, insulin resistance being the most reproducible factor in the development of NAFLD.^16^

NAFLD patients typically present with mild to moderate elevations of serum transaminase levels reflecting either hepatic injury or inflammation.^17^ The positive ultrasonographic correlation of the increasing severity of fatty liver changes with the increasing liver enzyme levels of the subjects indicate a progressive type of liver damage. While the elevation of liver enzymes has multiple causes, the methodology excluded other possible confounding factors. In the study by Adibi et al they noted that liver enzyme levels significantly correlated with angiographic atherosclerosis independent of serum C reactive protein and the metabolic syndrome.^18^

In our study the degree of cardiovascular risk as estimated by the FRS was correlated with increasing fatty liver grade. There has been an increasing body of evidence that NAFLD is likely to be associated with increased CVD risk, and raises the possibility that NAFLD may be not only a marker but also an early mediator of atherosclerosis.^19^ Treeprasertsuk et al followed up 309 patients with NAFLD and noted a sensitivity of 80% and specificity of 74% in predicting 10 year risk of coronary artery disease.^20^ Another study showed that nonalcoholic fatty liver disease is an independent predictor of cardiovascular risk. ^21^ This may be due to the fact that aside from insulin resistance, NAFLD and atherosclerosis share many unifying mechanisms involving adipokines, proinflammatory and thrombogenic factors.^22^

We had a number of limitations in the study. First is that the diagnosis of NAFLD was based on ultrasonography and not confirmed histologically by liver biopsy. Ultrasound is a highly operator dependent procedure with significant interobserver and intraobserver variability.^23^ Due to invasiveness, risk of complications, and high cost, liver biopsy is neither feasible nor cost effective in the diagnosis and monitoring of NAFLD. Secondly, the study has a relatively small sample size of participants especially various grades of fatty liver, as it is based from a single local tertiary center may not necessarily be an adequate representation of the general population. Thirdly, the study design is cross sectional and may not be fully adequate to confirm associations with variables. Larger prospective studies with control groups and longer follow-up may be needed to establish the validity of these results and to find a better understanding of cardiovascular risk among NAFLD subjects.

In that regard, ultrasound is a non-invasive, low risk, easy to use, readily available, and relatively low cost procedure that can reliability detect the presence of NAFLD. It can potentially be used as a routine screening and diagnostic tool for at risk patient populations. This study adds knowledge to the growing evidence that NAFLD may be a new and important cardiovascular risk factor.^24^ Pending additional research, the presence of NAFLD may be useful in the risk stratification of patients. This has potential therapeutic and prognostic implications as the presence of NAFLD may help identify patients who can benefit from early and aggressive therapies, both pharmacological as well as lifestyle modifications in order to improve outcomes.

## Conclusion

Ultrasound-based grading of the severity of NAFLD is associated with abnormalities in the metabolic profiles of patients. The FRS is correlated with increasing severity of NAFLD based on ultrasound. The presence of NAFLD may help identify those patients who are expected to derive the most benefit from aggressive primary intervention to prevent cardiovascular events.

## Ethical approval

The Institutional Ethics Review Board approved the study.

## Competing interests

None.

## Acknowledgments

The authors would like to thank the Makati Medical Center Radiology Department Ultrasound Section, the Makati Medical Center Corporate Health and Wellness Section and the Makati Medical Center Medical Records Section for their cooperation in the completion of the study.
